# One end to rule them all: Non-homologous end-joining and homologous recombination at DNA double-strand breaks

**DOI:** 10.1259/bjr.20191054

**Published:** 2020-02-28

**Authors:** Michael Ensminger, Markus Löbrich

**Affiliations:** 1 Radiation Biology and DNA Repair, Technical University of Darmstadt, 64287 Darmstadt, Germany

## Abstract

Double-strand breaks (DSBs) represent the most severe type of DNA damage since they can lead to genomic rearrangements, events that can initiate and promote tumorigenic processes. DSBs arise from various exogenous agents that induce two single-strand breaks at opposite locations in the DNA double helix. Such two-ended DSBs are repaired in mammalian cells by one of two conceptually different processes, non-homologous end-joining (NHEJ) and homologous recombination (HR). NHEJ has the potential to form rearrangements while HR is believed to be error-free since it uses a homologous template for repair. DSBs can also arise from single-stranded DNA lesions if they lead to replication fork collapse. Such DSBs, however, have only one end and are repaired by HR and not by NHEJ. In fact, the majority of spontaneously arising DSBs are one-ended and HR has likely evolved to repair one-ended DSBs. HR of such DSBs demands the engagement of a second break end that is generated by an approaching replication fork. This HR process can cause rearrangements if a homologous template other than the sister chromatid is used. Thus, both NHEJ and HR have the potential to form rearrangements and the proper choice between them is governed by various factors, including cell cycle phase and genomic location of the lesion. We propose that the specific requirements for repairing one-ended DSBs have shaped HR in a way which makes NHEJ the better choice for the repair of some but not all two-ended DSBs.

## Introduction

Among the various damages to the DNA molecule, a double-strand break (DSB) stands out because it disrupts both strands of the DNA double helix in close proximity. This essentially cuts the DNA into two parts and generates a two-ended DSB. Such DSBs arise endogenously by various processes and can be induced by exogenous agents such as ionizing radiation (IR), topoisomerase II inhibitors, and several radiomimetic drugs.^1,2^

Two main pathways exist for repairing two-ended DSBs in mammalian cells, non-homologous end-joining (NHEJ) and homologous recombination (HR). NHEJ, arguably the simpler of the two pathways, uses minimal processing of the break ends prior to rejoining them. It involves the end-binding heterodimer Ku70/Ku80 as well as the catalytic subunit of the DNA-dependent protein kinase, DNA-PKcs, constituting together with Ku70/Ku80 the DNA-PK holoenzyme. This complex limits DNA end processing and facilitates the recruitment of the downstream NHEJ factors XRCC4, XLF, and DNA ligase IV, which mediate the rejoining process.^3^ Unlike NHEJ, HR uses homologous sequences elsewhere in the genome to retrieve sequence information that was lost at the break site. HR starts with resection of the break ends, leading to RPA-coated single-stranded overhangs. Brca2 subsequently replaces RPA with Rad51 to form what is called a Rad51 nucleoprotein filament. Such a filament can pair to homologous sequences somewhere else in the genome and form a displacement loop (D-loop) followed by DNA repair synthesis to retrieve sequence information from the donor. During this process, a joint molecule between the broken DNA and the donor homologous template is formed. Different subpathways of HR exist and separate such joint molecules through distinct mechanisms.^2,4,5^

One-ended breaks, in contrast to two-ended DSBs, arise from replication problems. This can involve stalling of a fork at a replication-blocking lesion followed by replication fork collapse and breakage of one of the two sister chromatids formed behind the replication fork. A one-ended break can also arise when the replication machinery encounters a single-strand break and, upon unwinding of the DNA at the fork site, causes the disconnection of one of the two chromatids.^2,6–8^ The repair of one-ended breaks is arguably more difficult than the repair of two-ended DSBs and represents a particular challenge for the mechanisms devoted to maintaining genome stability.

Since a normal replication fork cannot be rebuilt at a collapsed or broken replication site, the classical semi-conservative mode of DNA replication cannot proceed. Instead, cells are able to employ a specialized HR subpathway termed break-induced replication (BIR) to resume DNA replication.^9–11^ This process involves the annealing of a broken end containing a single-stranded overhang to a single-stranded gap on the unbroken molecule. This step can be considered conceptually analogous to the Rad51-mediated step of D-loop formation during classical HR but appears to involve the strand annealing factor Rad52 instead of a Rad51 filament.^12^ Replication is resumed by a conservative mode of DNA synthesis where one chromatid contains both newly synthesized strands.^13,14^ In addition to BIR, one-ended DSBs can also be repaired by classical HR and possibly even by end-joining pathways if the second end is generated by an approaching replication fork.^15^ This, however, would need regulatory mechanisms to temporally coordinate the repair process with the progression of the cell cycle. Here, we discuss recent findings of how cells regulate the processes of NHEJ and HR at two-ended DSBs and elaborate on ideas about repair pathway usage at one-ended DSBs.

### NHEJ and HR both repair two-ended DSBs

**Figure 1.**
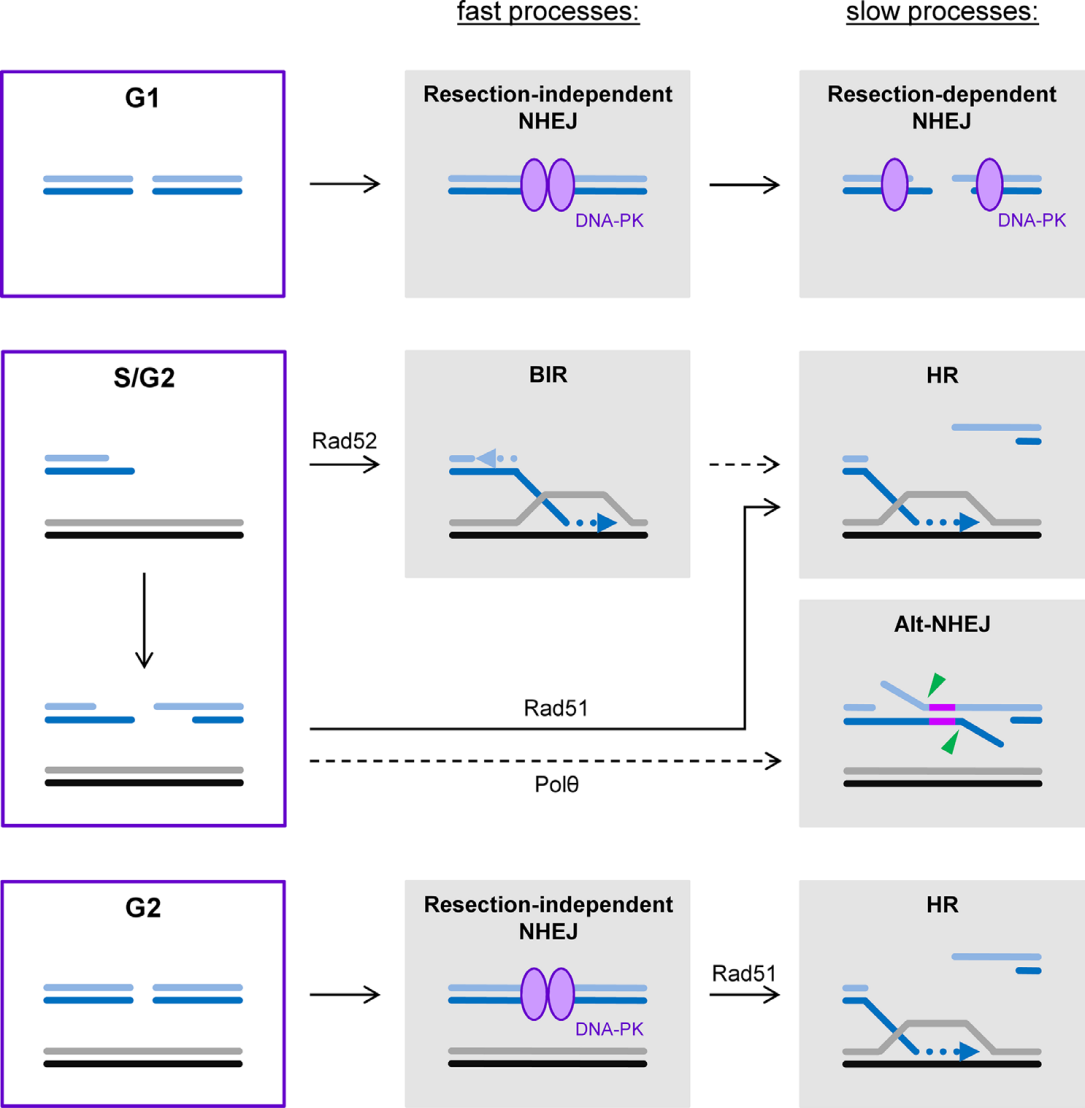
DSB repair pathways throughout the cell cycle. The majority of two-ended DSBs in G1 phase are repaired by the fast process of resection-independent NHEJ. The remaining DSBs undergo limited end-resection, allowing slow repair by resection-dependent NHEJ. In S phase, one-ended DSBs arise from replication problems and can be repaired by the specialized HR subpathway BIR. Arguably more often, however, a second break end is generated by an approaching replication fork, converting the one-ended into a two-ended DSB that can be repaired by classical HR pathways with Polθ-dependent alt-NHEJ serving as a backup pathway in the absence of functional HR. The BIR process might also involve the engagement of a second break end. Similar to G1 phase, the majority of two-ended DSBs in G2 are repaired by resection-independent NHEJ. However, resection of the remaining DSBs is extensive allowing repair by HR.

The pathways for repairing two-ended DSBs are best studied by analyzing cells maintained in G1 or G2 during repair since this prevents the formation of one-ended DSBs during replication.^16^ Earlier studies with confluent cell cultures revealed that IR-induced DSBs are repaired with two-component kinetics, involving a fast process within the first few hours followed by a slower process extending over many hours after damage induction.^17^ The analysis of mutant cells showed that both processes require the classical NHEJ factors but the slow process additionally involves the factors ATM, Artemis and proteins locating to γH2AX foci.^18,19^ Subsequent studies uncovered that the slow repair process requires ATM-mediated chromatin remodeling and involves a limited amount of end-resection.^20,21^ This resection step in G1/G0 cells utilizes some of the same resection factors employed during HR but has distinct features that allow the rejoining of the break ends by the NHEJ machinery.^22,23^ The fast and the slow NHEJ process also differ in their risk to form genomic rearrangements from the joining of incorrect break ends. While rearrangements occur fairly infrequently during the fast process, they arise about 5-fold more often from the slow process.^22,24^ The higher propensity to form rearrangements likely results from the increased mobility of slowly repairing DSBs.^25^ It is currently unknown why some breaks are repaired by fast NHEJ without resection while others undergo resection and slow NHEJ. The requirement for ATM suggests that the chromatin environment is an important factor but the chemical complexity of a DSB also favors slow over fast NHEJ.^18,26^ Perhaps the most intuitive model is that cells first try to repair DSBs fast and without major end-modifications and only employ a more sophisticated resection program if the breaks reside in specific genomic locations or contain chemical end-structures that preclude fast repair (Figure 1).

In contrast to G1, cells irradiated in G2 employ HR in addition to NHEJ for the repair of two-ended DSBs. This suggests that a sister chromatid serves as the template for repair during HR, as opposed to the homologous chromosome which is also present in G1 cells where repair exclusively proceeds by NHEJ. Repair in G2 exhibits similar two-component kinetics as in G1 where the fast repair process also involves the classical NHEJ factors but the slow process represents HR (Figure 1).^27^ This slow process also requires ATM-mediated chromatin remodeling and a resection step involving Artemis but orchestrates it in a manner compatible with the formation of a Rad51 nucleoprotein filament, a prerequisite for homology search and HR.^28^ Collectively, this analysis showed that NHEJ without resection constitutes the fast repair process both in G1 and G2 and repairs the majority of IR-induced DSBs. We have termed this pathway “resection-independent NHEJ”. The slow process involves resection in both cell cycle phases, albeit to a different degree and regulated in a different manner. We have termed the slow NHEJ process in G1 “resection-dependent NHEJ” (Figure 1).^3,29^ Why NHEJ is utilized not only in G1 but also in G2 when a sister chromatid can serve as a template for repair by HR is an open question which is discussed below.

### HR repairs one-ended DSBs but end-joining can do so too

The repair of a one-ended DSB represents a particular challenge since both major DSB repair pathways, NHEJ and HR, rely on connecting two break ends, either without or with the potential to restore the sequence information that was lost at the break site. Moreover, broken replication forks, from which one-ended DSBs arise, cannot be rebuilt to resume replication since the required components such as the replication pre-initiation complex are no longer available.^15,30^ It appears that lower, and to a certain extent also higher, eukaryotes have solved this problem by the development of BIR (Figure 1).^9–11^ How this process terminates in mammalian cells is largely unknown but likely involves the encounter of the BIR site with an approaching replication fork. At the site of such an encounter, both replication structures might converge and form a complex that needs processing to finalize the repair process. It is possible that this complex has similarities with the joint molecule structures arising during the classical HR pathway(s). In any case, the process of BIR, if not extended to the end of the chromosome,^31^ would entail the involvement of a second replication site and possibly the generation of a second break end. Thus, the processes for repairing one-ended DSBs might encompass many of the same concepts that apply to the repair pathways for two-ended DSBs.

Insight into DSB repair pathway usage at replication-associated one-ended DSBs largely comes from studies with genotoxic agents inducing base damages or single-strand breaks. Such lesions, if encountered by the replication fork, can generate one-ended DSBs upon stalling and collapse of the forks.^6^ Indeed, treatment with the alkylating agent methyl methanesulfonate causes DSBs during replication whose repair depends on the HR pathway.^32^ Likewise, chromosome aberration formation is substantially enhanced in HR mutants compared with wt cells or NHEJ mutants.^33^ The predominant role of HR for repairing one-ended DSBs is further demonstrated by the exquisite sensitivity of HR mutants to a variety of agents that induce single-stranded DNA lesions, including the topoisomerase I inhibitor camptothecin.^34–36^ Finally, the majority of spontaneous DSBs arise at replication forks, likely from endogenously arising single-stranded DNA lesion, and necessitate a functional HR pathway to provide cell survival.^32^ Thus, the prevailing evidence suggests that HR represents the predominant pathway for repairing one-ended DSBs.^2,5^ However, it is important to note that an alternative NHEJ (alt-NHEJ) pathway dependent on polymerase θ (Polθ) can also repair resected DSBs in the absence of HR and is important for cell survival in HR mutant tumor cells.^37^ Collectively, this suggests that one-ended DSBs arising at replication forks are converted into two-ended DSBs that are predominantly repaired by HR with alt-NHEJ serving as a backup pathway in the absence of functional HR (Figure 1).

### Regulating HR to prevent end-joining of resected breaks

**Figure 2.**
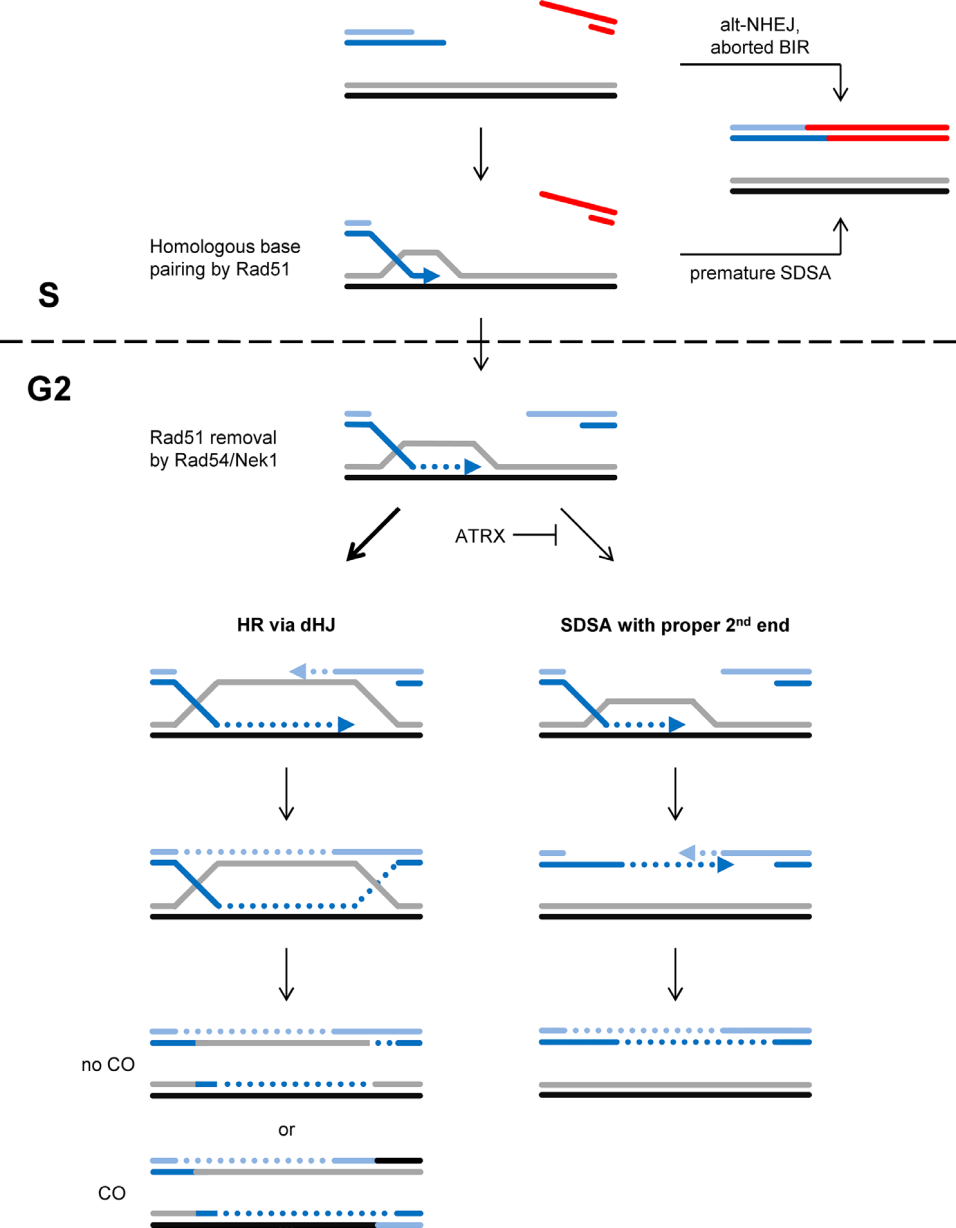
HR at one-ended DSBs. One-ended DSBs are repaired by HR processes, likely using a second break end generated from an approaching replication fork. HR is initiated by Rad51-mediated base pairing of the resected break end to a homologous sequence. For DNA repair synthesis and HR to proceed, Rad51 needs to be removed by Rad54, a step which is postponed until G2 phase due to the G2-specific activation of Rad54 by Nek1. This ensures that DNA repair synthesis starts at a time when the second break end is available. The dHJ subpathway of HR involves second-end capture before processing the joint molecules, providing an intrinsic feature to control for the availability of a second break end. SDSA, in contrast, involves the displacement of the synthesized strand from the homologous donor, a step which could occur before the second end is created and bears the risk to join break ends from different DSBs. Joining incorrect break ends can also occur if one-ended DSBs are repaired by alt-NHEJ or by BIR processes that are aborted before a second break end is available. The choice between the dHJ pathway and SDSA is regulated by the chromatin remodeler ATRX.

The two main subpathways of HR are synthesis-dependent strand annealing (SDSA) and a pathway involving the formation of double Holliday junctions, henceforth referred to as the dHJ pathway.^4^ Following D-loop formation and DNA repair synthesis, SDSA proceeds by the displacement of the synthesized strand from the donor molecule and annealing with the second DSB end that did not engage in homology search and strand invasion (Figure 2).^38^ The dHJ pathway, in contrast, involves the annealing of the second, non-invading DSB end to the D-loop, a step called second-end capture. This forms a structure that has been suggested to represent two crossing points between the two participating molecules that are termed Holliday junctions. Upon resolution of these junctions, cross-overs (COs) between the molecules can arise.^38,39^ In case a DSB is repaired by HR using a sister chromatid as a template, such COs will manifest as sister chromatid exchanges (SCEs) which are genetically neutral since both sister chromatids contain the exact same genetic information (Figure 2).^4,40^ However, if HR involves the homologous chromosome or a homologous sequence on a heterologous chromosome, a CO event leads to loss of heterozygosity or the formation of chromosomal rearrangements.^5,38^ Thus, it has been suggested that cells limit the dHJ pathway to meiosis when recombination between the homologous chromosomes is desirable and employ SDSA for the repair of DSBs arising in mitotically growing cells.^4^

We have recently shown that the chromatin remodeler ATRX promotes an HR subpathway that involves extended DNA repair synthesis and the formation of SCEs, suggesting that this pathway represents the dHJ process. Indeed, ATRX limits the usage of SDSA which has been suggested to involve only short patches of DNA repair synthesis and no SCE formation.^41,42^ So why do cells use an HR subpathway which forms COs when SDSA appears to be the safer means? An answer to this question may lie in the consideration that spontaneously arising DSBs harbor only one end. As discussed above, it is likely that the second break end needed for repair by HR will be generated from an approaching replication fork. It might therefore be beneficial for a cell to employ an HR pathway which involves second-end capture before the joint molecule between the broken DNA and the donor homologous template is processed. This would not be the case for SDSA where displacement of the synthesized strand from the donor molecule likely occurs irrespective of the availability of a second end. Indeed, strand displacement in the absence of a second break end harbors the risk of annealing one-ended DSBs from different genomic regions, resulting in deleterious genomic rearrangements (Figure 2). Thus, we suggest that one reason for the preferential usage of the dHJ pathway over SDSA might be that HR has evolved to repair one-ended DSBs at collapsed replication forks where premature displacement of the synthesized strand carries the risk of rearrangement formation. If processing of the joint molecule follows on a second-end capture step, as is the case for the dHJ pathway, rearrangement formation is limited. This advantage appears to come at the costs of forming COs which, however, are genetically neutral as long as HR is restricted to the sister chromatid and does not involve another chromosome (Figure 2).^5^

Another finding about the regulation of HR might also be viewed in the context of HR having evolved to repair one-ended DSBs. As introduced above, HR involves the formation of a Rad51 nucleoprotein filament pairing to its homologous template. For DNA repair synthesis to start, Rad51 is removed with the help of Rad54.^43–45^ We recently showed that this function of Rad54 requires its phosphorylation by Nek1 which, unexpectedly, occurs in the late G2 phase of the cell cycle even if DSBs arise during S phase.^46^ We suggest that this delay of DNA repair synthesis serves to postpone later HR stages until a second break end has been generated from an approaching replication fork. This provides the possibility for second-end capture and minimizes the chances for strand displacement and rearrangement formation in the absence of a second end (Figure 2).

Collectively, recent findings suggest that the process of HR may have evolved to repair one-ended DSBs. Such lesions are likely converted into two-ended DSBs by approaching replication forks and HR appears to be regulated to minimize the potential for joining break ends from different DSBs. Such regulatory mechanisms include the delay in DNA repair synthesis until very late phases of the cell cycle and a second-end capture step prior to the processing of the joint molecules.^41,42,46^ The second-end capture step likely leads to the formation of dHJs which, upon resolution, can form COs. Since COs can be deleterious if a repair template other than the sister chromatid is used, this provides another explanation of why HR is restricted to post-replicative cell cycle phases.^5^

### So why not always use HR in G2?

**Figure 3.**
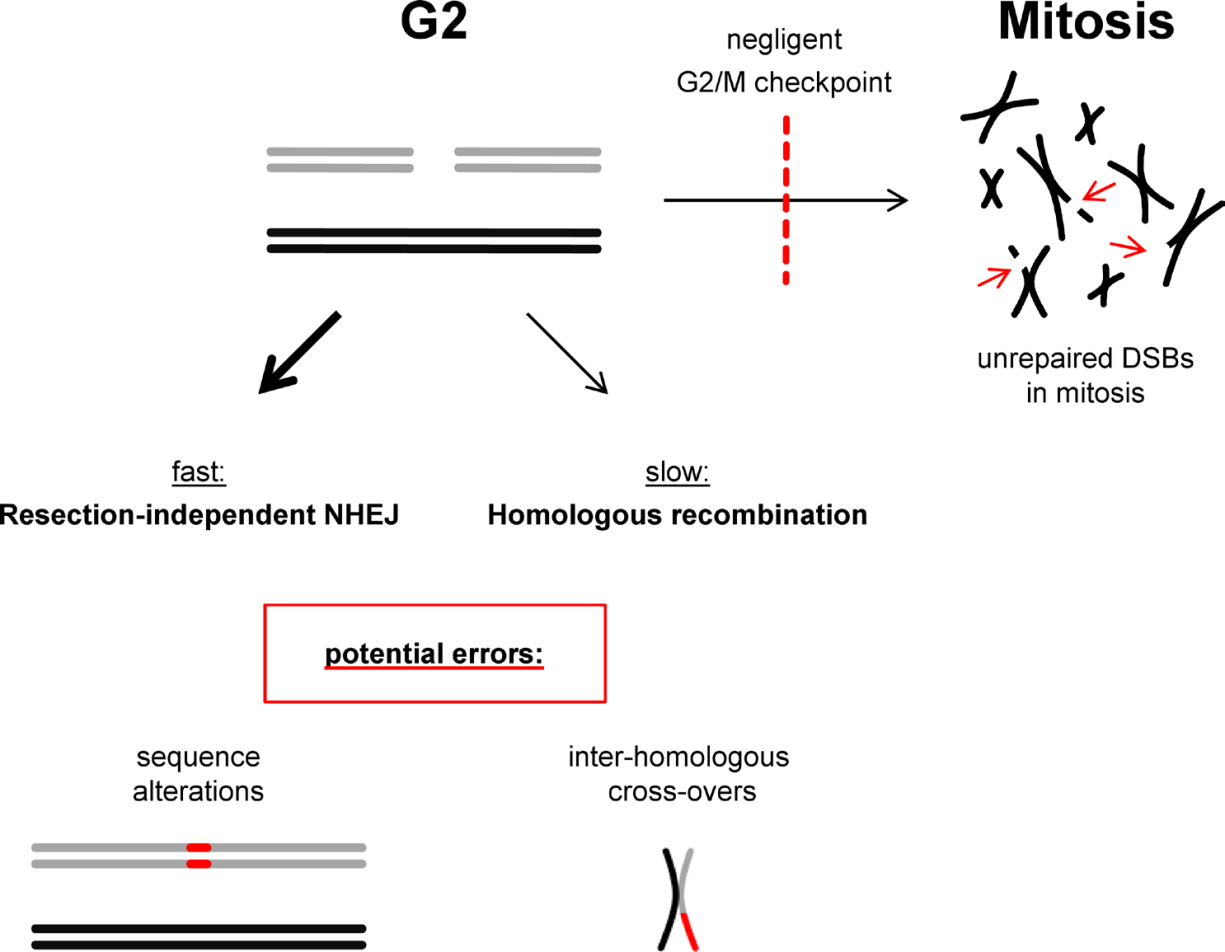
NHEJ and HR at two-ended DSBs in G2. Two-ended DSBs arising in G2 phase can be repaired by fast resection-independent NHEJ and slow HR. NHEJ is potentially error-prone since it cannot reconstitute sequence information that is lost at the break site. HR is error-free if the homologous sequence on the sister chromatid is used. However, HR can cause COs which are deleterious if it involves a homologous template other than the sister chromatid or if the sister chromatid is used off-frame, *e.g*. at repetitive sequences. Thus, both processes are potentially error-prone. Moreover, the G2/M checkpoint which serves to provide time for repair is negligent and allows cells to progress into mitosis with unrepaired DSBs. Thus, it might be beneficial for a cell to use fast NHEJ instead of slow HR to repair as many breaks as possible.

As outlined above, both NHEJ and HR repair two-ended DSBs that arise in G2 phase, where NHEJ represents the fast and HR the slow repair component.^27,28^ Our understanding about the regulatory mechanisms of HR might help to answer the question of why HR is not exclusively used for such lesions. The necessity to temporally coordinate HR with the generation of a second break end at a collapsed replication fork requires the delay of DNA repair synthesis until very late phases of the cell cycle.^46^ Moreover, second-end capture is employed to prevent the premature processing of joint molecules before a second break end is available. This results in the formation of dHJs which are resolved in a manner generating COs. Thus, despite being considered error-free, HR has the potential to generate rearrangements if a template other than the sister chromatid is used or if the sister chromatid is used off-frame at repetitive regions.^38^ NHEJ, on the other hand, is likely to join correct break ends if employed quickly after DSB induction and might only carry a significant risk for joining incorrect ends for DSBs that are refractory to fast repair.^22,24^ Moreover, since the G2/M checkpoint is negligent and allows the progression of cells with unrepaired DSBs into mitosis,^47–49^ it might simply be the better option to repair as many DSBs as possible by fast NHEJ before engaging into the slower HR process which also has its limitations (Figure 3).

## Conclusion

The intricate choice between employing NHEJ or HR for DSB repair might be largely governed by the distinct risks of these pathways to form genomic rearrangements. NHEJ mostly rejoins correct break ends but rearrangements can arise from this pathway, particularly from the slow resection-dependent NHEJ process. HR, in contrast, is often regarded as being error-free. However, the consideration that the repair of one-ended DSBs demands an HR subpathway that engages a second break end and forms COs reveals the limitations of HR. CO formation is genetically neutral if the homologous template on the sister chromatid is used but leads to rearrangements in all other cases. This explains why cells favor resection-dependent NHEJ over HR in G1 when no sister chromatid is available. It also elucidates why the fast resection-independent NHEJ process is employed together with HR in G2, particularly in light of the short duration of this cell cycle phase and the negligence of the G2/M checkpoint.
